# Complex coupled metabolic and prokaryotic community responses to increasing temperatures in anaerobic marine sediments: critical temperatures and substrate changes

**DOI:** 10.1093/femsec/fiv084

**Published:** 2015-08-04

**Authors:** Erwan G. Roussel, Barry A. Cragg, Gordon Webster, Henrik Sass, Xiaohong Tang, Angharad S. Williams, Roberta Gorra, Andrew J. Weightman, R. John Parkes

**Affiliations:** 1 School of Earth and Ocean Sciences, Main Building, Park Place, Cardiff University, CF10 3AT Cardiff, UK; 2 Cardiff School of Biosciences, Main Building, Park Place, Cardiff University, CF10 3AT Cardiff, UK; 3 DISAFA, University of Turin, Largo P. Baccini 2, 10095 Grugliasco, TO, Italy

**Keywords:** sediment, temperature, anaerobic processes, chemoorganotrophic, chemolithotrophic, mineralisation, sulphate reduction, methanogenesis, acetogenesis

## Abstract

The impact of temperature (0–80°C) on anaerobic biogeochemical processes and prokaryotic communities in marine sediments (tidal flat) was investigated in slurries for up to 100 days. Temperature had a non-linear effect on biogeochemistry and prokaryotes with rapid changes over small temperature intervals. Some activities (e.g. methanogenesis) had multiple ‘windows’ within a large temperature range (∼10 to 80°C). Others, including acetate oxidation, had maximum activities within a temperature zone, which varied with electron acceptor [metal oxide (up to ∼34°C) and sulphate (up to ∼50°C)]. Substrates for sulphate reduction changed from predominantly acetate below, and H_2_ above, a 43°C critical temperature, along with changes in activation energies and types of sulphate-reducing *Bacteria*. Above ∼43°C, methylamine metabolism ceased with changes in methanogen types and increased acetate concentrations (>1 mM). Abundances of uncultured *Archaea*, characteristic of deep marine sediments (e.g. MBGD *Euryarchaeota*, ‘*Bathyarchaeota’*) changed, indicating their possible metabolic activity and temperature range. Bacterial cell numbers were consistently higher than archaeal cells and both decreased above ∼15°C. Substrate addition stimulated activities, widened some activity temperature ranges (methanogenesis) and increased bacterial (×10) more than archaeal cell numbers. Hence, additional organic matter input from climate-related eutrophication may amplify the impact of temperature increases on sedimentary biogeochemistry.

## Abstract (precis)

Temperature increases had a non-linear effect on biogeochemistry and prokaryotes in marine sediments, with rapid changes over small temperature intervals which are relevant to climate change and the deep biosphere.

## Graphical abstract

**Graphical Abstract Figure.**
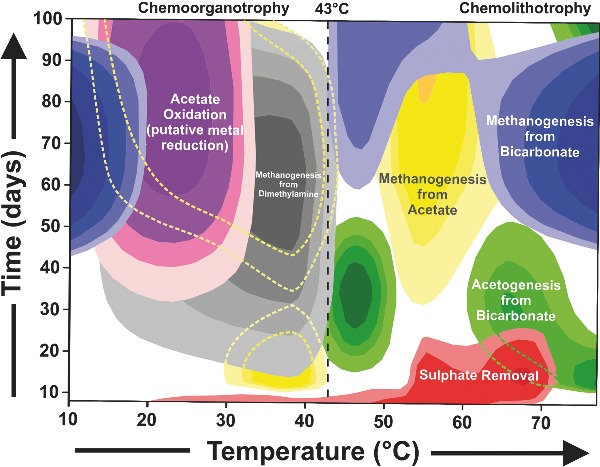
Temperature increases had a non-linear effect on biogeochemistry and prokaryotes in marine sediments, with rapid changes over small temperature intervals which are relevant to climate change and the deep biosphere.

## INTRODUCTION

Between 5 and 10 billion tons of particulate, organic matter are constantly sinking in the world's oceans and accumulating as sediments (Jørgensen 1983). Anaerobic microbial processes play a major role in the degradation of this organic matter, especially in coastal sediments (Canfield *et al.* 1993), with metal oxide and sulphate reduction being major degradation processes. The small amount of organic matter that is not degraded builds up over geological time to become the largest global store of organic carbon (Hedges and Keil 1995), which under some circumstances forms oil and gas after maturation and heating. This burial of reduced carbon also has a major impact on the surface environment in terms of the unused oxygen that accumulates in the atmosphere and removal of nutrients and other compounds. Sedimentary anaerobic communities, therefore, have a major impact on both the biosphere and geosphere.

These anaerobic communities involve interdependent, interacting groups of organisms, including hydrolytic/fermenters, heterotrophic acetogens, syntrophs and terminal-oxidizing groups, such as nitrate, metal oxide and sulphate reducers and methanogens. Although the concentration and turnover of metabolic intermediates between these groups (e.g. H_2_ and acetate) can provide some information about the dominant processes and their interactions (Lovley and Chapelle 1995; Parkes *et al.* 2007a), as can inhibitor (Parkes *et al.* 1989) and biodiversity studies (Fry *et al.* 2008), there is still only limited information about the interactions between a range of different anaerobic processes and how these are influenced by environmental conditions. The response of some anaerobic processes to changing conditions cannot be adequately explained by thermodynamic considerations (Rothfuss and Conrad 1993; Peters and Conrad 1996); hence, other approaches are also needed. Temperature changes have been used to alter the dominant anaerobic pathways and associated prokaryotic populations (Conrad, Klose and Noll 2009) or uncouple key phases in organic matter degradation, to reveal metabolic interactions (Weston and Joye 2005; Finke and Jørgensen 2008). Often, substrates are added to these experiments to ensure elevated microbial activity, to allow changes to be clearly seen and/or to relieve terminal oxidizers from substrate limitation by fermenters/syntrophs, which might have different metabolic/temperature controls. Using these types of experiments with coastal marine sediments, Weston and Joye (2005) showed that hydrolysis/fermentation was enhanced at low temperatures, <25°C, resulting in an accumulation of organic acids, whilst >25°C sulphate reduction was initially enhanced resulting in net removal of organic acids which ultimately limited sulphate reduction.

Similarly, it has been shown that in anoxic rice field soils acetate concentrations increased with decreasing temperatures, from about 5 μM between 17 and 37°C to about 50 μM at 10°C, and acetoclastic methanogenesis becomes increasingly dominant (Fey and Conrad 2000). The activity of psychrotolerant heterotrophic acetogens was suggested as an explanation for this effect. At higher temperatures, methanogenesis changed from a mixture of acetoclastic and hydrogenotrophic methanogenesis to exclusively hydrogenotrophic methanogenesis over a surprisingly narrow temperature range of 42–46°C (Conrad, Klose and Noll 2009). These studies suggested that temperature defined the structure and function of the methanogenic community in anoxic rice field soils. At temperatures above ∼50°C, in rice field soils (Rui, Qiu and Lu 2011), anaerobic digesters (Ho, Jensen and Batstone 2013) and oil reservoir fluids (Dolfing, Larter and Head 2008; Mayumi *et al.* 2011) data also suggests dominance of hydrogenotrophic methanogenesis, but that this is coupled to acetate oxidation by syntrophs producing H_2_.

In contrast to the above, Finke and Jørgensen (2008) concluded that in temperate marine sediments fermentative bacteria tolerated higher temperatures than terminal oxidizing, sulphate-reducing bacteria, and hence, above a critical temperature of ∼30°C concentrations of organic acids and H_2_ increased. Subsequent removal of H_2_ in these experiments suggested that methanogens also tolerated higher temperatures than sulphate-reducing bacteria. This is surprising as several studies have shown that thermophilic, spore-forming sulphate-reducing bacteria are present in coastal sediments and become active at these higher temperatures (Isaksen, Bak and Jørgensen 1994; Muller *et al.* 2014; O'Sullivan *et al.* 2015).

Despite differences between studies, the above clearly shows that varying temperatures can result in the dominance of different anaerobic prokaryotic processes and can help determine the nature of the interactions between both competing and complementary processes. There is also a suggestion of critical temperatures, where changes in function and community structure occur over a surprisingly narrow temperature range (e.g. Conrad, Klose and Noll 2009). To explore this further, we investigated the impact of incubating temperate estuarine sediments at a wide range of different temperatures (0–80°C) for up to 100 days, either with (1) a small addition of H_2_ to stimulate activity and interactions (e.g. via the impact of sulphate depletion) or (2) a significant substrate addition to potentially highlight the temperature impact on terminal oxidizers separate from their substrate suppliers, and to increase their population size, and hence, their interaction and detection. Geochemical and direct radiotracer analyses were used to measure biogeochemical activities, and community composition was determined by 16S rRNA gene analysis. This was complemented by thermodynamic and kinetic analysis.

## MATERIALS AND METHODS

### Sampling and sediment slurries

Sediment slurries were prepared from sediment cores, depths to 49–58 cm, collected at low tide from tidal flats of the Severn Estuary, Woodhill Bay, Portishead, UK (51°29^′^31.66^″^ N, 2°46^′^27.95^″^ W) on 12 February 2010 using Plexiglas core tubes. At high tide, the sediment was covered by ∼1.5 m of water. The winter *in situ* sediment temperature was low, 6.8°C compared to average local sea surface temperature (mean: 12.6°C, range 3.6–22.6°C; Joyce 2006). After sampling, cores were sealed with rubber bungs and brought back to the laboratory within two hours for rapid geochemical processing. To assess the effect of seasonality on the geochemical profiles, data were compared to a summer (June) geochemical depth profile analysed under identical conditions (*in situ* temperature 18.7°C).

To avoid high sulphate concentrations and thus the potential dominance of sulphate reduction, and inhibition of methanogenesis and/or acetogenesis, only sediment below 30 cm, which also contained methane, was slurried (Fig. S1, Supporting Information). All sediments were thoroughly homogenized in a gas-tight plastic bag under oxygen-free nitrogen, and then added to modified 2 L screw-capped bottles (1:4, v/v) containing anoxic mineral salts medium reduced with 1 mM sodium sulphide (Wellsbury, Herbert and Parkes 1994), and the gas headspace replaced with N_2_:CO_2_ (80:20, v/v). Slurries were incubated at 10°C (∼*in situ* annual average temperature) on an orbital shaker (100 rpm) in the dark until sulphate concentration reached steady state and sediment was homogeneously slurried. Replicate slurries were then distributed in an anaerobic cabinet into either 20 mL (10 mL slurry) or 60 mL serum vials (20 mL slurry), and sealed with butyl rubber septa. Half of the slurries were amended with 2 mM acetate and 2 mM methylamine prior to dispensing and then had their headspace gas replaced by H_2_:CO_2_ (80:20, v/v); these were the substrate-amended vials. No substrates were added to the other slurries, but they were left with a small amount of H_2_ (∼45 μM) from the anaerobic cabinet to slightly stimulate prokaryotic activity and interactions; these were termed unamended slurries. Five series of 24 substrate-amended and unamended 20 mL slurry vials were incubated upside down between 0 and 80°C in a Thermal Gradient System (Parkes *et al.* 2007b). Each series was then sacrificed at different time points, up to 100 days, for analysis. The 60 mL serum vials, which contained only unamended slurry, were incubated upside down in the dark at 10, 25, 38, 46, 55, 66 and 77°C for ^14^C-activity measurements. Additional aliquots of both slurry types (20 mL) were incubated at the same temperatures, as ‘indicator vials’, whose headspace gases were repeatedly sampled over time to help determine the appropriate sampling times for the thermal gradient vials and ^14^C-activity measurement times.

### Pore water and gas analysis

Sediment and slurry headspace gases were analysed by a natural gas analyser (PerkinElmer Clarus^®^ 500) as previously described (Webster *et al.* 2010). Anion and cation concentrations from sediment and slurry pore waters were determined by ion chromatography (Dionex ICS-2000 and DX-120, Camberley UK; Webster *et al.* 2010). Dissolved metals in pore waters were analysed by Inductively-Coupled Plasma Mass Spectrometer (ICP-MS) as previously described (Moreno *et al.* 2007).

### Activity rate measurements

Hydrogenotrophic methanogenesis, hydrogenotrophic acetogenesis, acetoclastic methanogenesis, methylotrophic methanogenesis and acetate oxidation rates were measured using ^14^C radiolabelled substrates ([1,2-^14^C]acetic acid, [^14^C]bicarbonate and [1,2–^14^C]dimethylamine; Parkes *et al.* 2007a, 2012). Each activity was measured at three increasing time periods in triplicate; data are expressed as an average of the three time-point means. Acetogenesis was measured by collection of the ^14^C-acetate fraction using a Dionex ICS-2000 Ion Chromatography System equipped with a Foxy Jr.^®^ fraction collector (Teledyne Isco) followed by liquid scintillation counting. Rates of acetate oxidation to carbon dioxide were calculated by multiplying rates by 2 to account for the two carbon dioxide molecules generated from each acetate molecule. Sulphate removal rates were calculated from the difference in sulphate concentrations between each sampling time point.

### Thermodynamic calculations and Arrhenius parameters

The Gibbs free energy (ΔG’_r_) under non-standard conditions was calculated as previously described (Conrad and Wetter 1990). An estimation of the temperature dependence of each studied anaerobic process was obtained by calculating the activation energy (*Ea*) and the Q_10_ factor from Arrhenius plots (Aller and Yingst 1980). The Arrhenius profiles were obtained by plotting the natural logarithm of each maximum rate for each incubation temperature versus the inverse of temperature. The activation energy for each metabolic process was calculated from the following equation:

\begin{equation*}
\ln (k) = \ln (A) + \left( {\frac{{ - Ea}}{R}\cdot\frac{1}{T}} \right),
\end{equation*}

where *Ea* is the activation energy (kJ mol^−1^), *k* is the reaction rate (nmol cm^−3^ day^−1^), *A* is the Arrhenius constant, *R* is the gas constant (8.314 × J K^−1^ mol^−1^) and *T* is the absolute temperature (K). Q_10_ is the factor by which the rate of reaction increases with a temperature increase of 10°C. The selected temperature range in this study was between 10 and 20°C. Q_10_ was calculated using the following equation:

\begin{equation*}
Q_{10} = e^{\frac{{E_a }}{R} \cdot \frac{{\Delta T}}{{T_1 \cdot T_2 }}}
\end{equation*}

### Carbon dioxide balance

In order to compare chemoorganotrophic and chemolithotrophic processes, the total carbon dioxide generation rates were determined from the net production or consumption of carbon dioxide by each measured metabolic process. The carbon dioxide generation rate for each metabolic process studied was calculated by multiplying the metabolic rate for each incubation time and temperature by the factor described in Table S1 (Supporting Information). A standardizing factor of 2 was used for putative acetoclastic metal reduction.

### DNA extraction

Genomic DNA was extracted from sediment slurries using the FastDNA^®^ Spin Kit for Soil (MP Biomedicals) as described (Webster *et al.* 2003). Essentially, 3 mL (1.5 mL × 2) of sediment slurry was placed in a lysing matrix E tube (MP Biomedicals) and centrifuged at 15 000 × *g* for 1 min to pellet cells and sediment. Pellets were then resuspended in 800 μl of sodium phosphate buffer and 120 μl MT buffer (MP Biomedicals) before lysis in a FastPrep^®^ 24 instrument (MP Biomedicals) for 2 × 30 s at speed 5.5 m s^−1^. All remaining steps were as per the manufacturer's protocol, except that some spin and incubation times were extended. DNA was eluted in 100 μl molecular grade water (Severn Biotech Ltd.) and stored at –80°C until required.

### PCR-DGGE analysis of 16S rRNA genes

Bacterial and archaeal 16S rRNA genes were amplified by either direct or nested PCR from all sediment slurry DNA extracts using Dream*Taq* DNA polymerase (Thermo Fisher Scientific Inc.) with primers 357FGC/518R for *Bacteria* and 109F/958R followed by SAFGC/PARCH519R for *Archaea* as previously described (Webster *et al.* 2006; O'Sullivan *et al.* 2013). All 16S rRNA gene PCR products (ca. 200 ng of each product) were separated by DGGE on 6–12% gradient (w/v) polyacrylamide DGGE gels with a 30–60% denaturant gradient (Webster *et al.* 2006; O'Sullivan *et al.* 2013). DGGE gels were stained with SYBR Gold nucleic acid stain (Invitrogen), viewed under UV and images captured using a Gene Genius Bio Imaging System (Syngene). DGGE bands, representative of all major phylotypes, were excised, reamplified by PCR, sequenced (O'Sullivan *et al.* 2008) and band identity determined using the NCBI nucleotide BLAST program (http://www.ncbi.nlm.nih.gov/). All 16S rRNA gene sequences reported here have been submitted to the GenBank database under accession numbers KR632942–KR632979.

### Quantitative real-time PCR (qPCR)

qPCR was used to quantify 16S rRNA gene copy numbers of *Bacteria* and *Archaea* in sediment slurries. SybrGreen chemistry was used for all protocols. All qPCR reactions for standards, no template controls and sediment DNA samples were conducted in triplicate and run on an Agilent Mx3000P QPCR System (Agilent Technologies UK Ltd). For standard curves and calibration, serial dilutions of full length 16S rRNA gene PCR products from *Anaerolinea thermophila* DSM 14523 and *Methanococcoides methylutens* DSM 2657 were used as standards for *Bacteria* and *Archaea* (Webster *et al.* 2015). To ensure good quantification data, qPCR results were rejected if the *R*^2^ value of the standard curve was below 0.95 or the efficiency of the reaction was below 80%. The qPCR mixtures for all reactions (standards, controls and samples) were contained in a total volume of 20 μl with 400 nM of each primer (Eurofins MWG Operon), 2 μg bovine serum albumin (BSA; Promega) and 1 μl of DNA in 1× qPCRBIO SyGreen Lo-ROX Mix (PCR Biosystems Ltd) made up with molecular grade water (Severn Biotech Ltd). 16S rRNA gene primers 534F/907R and S-D-Arch-0025-a-S-17F/S-D-Arch-0344-a-S-20R were used to target the *Bacteria* and *Archaea*, respectively (Webster *et al.* 2015). The protocol was 95°C for 7 min, 40 cycles of 95°C for 30 s, 52°C for 30 s, 72°C for 60 s, followed by a melting curve from 55 to 95°C. Each cycle was followed by data acquisition at the elongation step.

To estimate the number of bacterial and archaeal cells, the 16S rRNA gene copy numbers were divided by the average 16S rRNA gene copy number for each taxa (4.19 and 1.71, respectively), deduced using the *rrn*DB database v3.1.225 (https://rrndb.umms.med.umich.edu/); (Stoddard *et al.* 2015). Average cell numbers and their standard deviations were calculated from each replicate.

### V3–V5 16S rRNA gene tag sequencing

Variable regions 3 to 5 (V4–V5) of the 16S rRNA gene from *Bacteria* and *Archaea* were amplified from a selected number of unamended slurry DNA samples covering a range of temperatures (25, 35, 38, 46, 66°C) and time points (0, 15, 35, 62 days) using barcoded fusion primers 357F/907R (Muyzer, Dewaal and Uitterlinden 1993; Muyzer *et al.* 1998) and 341F/958R (Delong 1992; Ovreas *et al.* 1997), respectively. All PCR reactions (in triplicate) and 454 pyrosequencing were performed by the Research and Testing Laboratory (Lubbock, TX, USA; http://www.researchandtesting.com/index.html) on a Roche 454 GS FLX Titanium system. A total of 126 608 *Bacteria* and 70 281 *Archaea* sequences were obtained. Analysis of sequencing data was performed in QIIME version 1.6.0 (Caporaso *et al.* 2010) using a pipeline developed ‘in house’ at Cardiff University. Essentially, all sequence files were checked using Acacia software release 1.53 (Bragg *et al.* 2012) for quality, sequence errors and to reduce noise. Chimeras were detected and removed using the USEARCH61 algorithm and each sample randomly subsampled to the lowest number of sequences in each library (2919 for *Bacteria* and 1617 for *Archaea*). Representative OTUs were picked with UCLUST (Edgar 2010) at 97% similarity and taxonomy assigned using BLAST (Altschul *et al.* 1990) with the Greengenes database (DeSantis *et al.* 2006). Singletons and non-specific sequences were then removed and diversity estimates were calculated in QIIME.

## RESULTS AND DISCUSSION

### Sediment geochemistry and the effect of different temperatures on anaerobic processes in unamended sediment slurries

**Figure 1.**
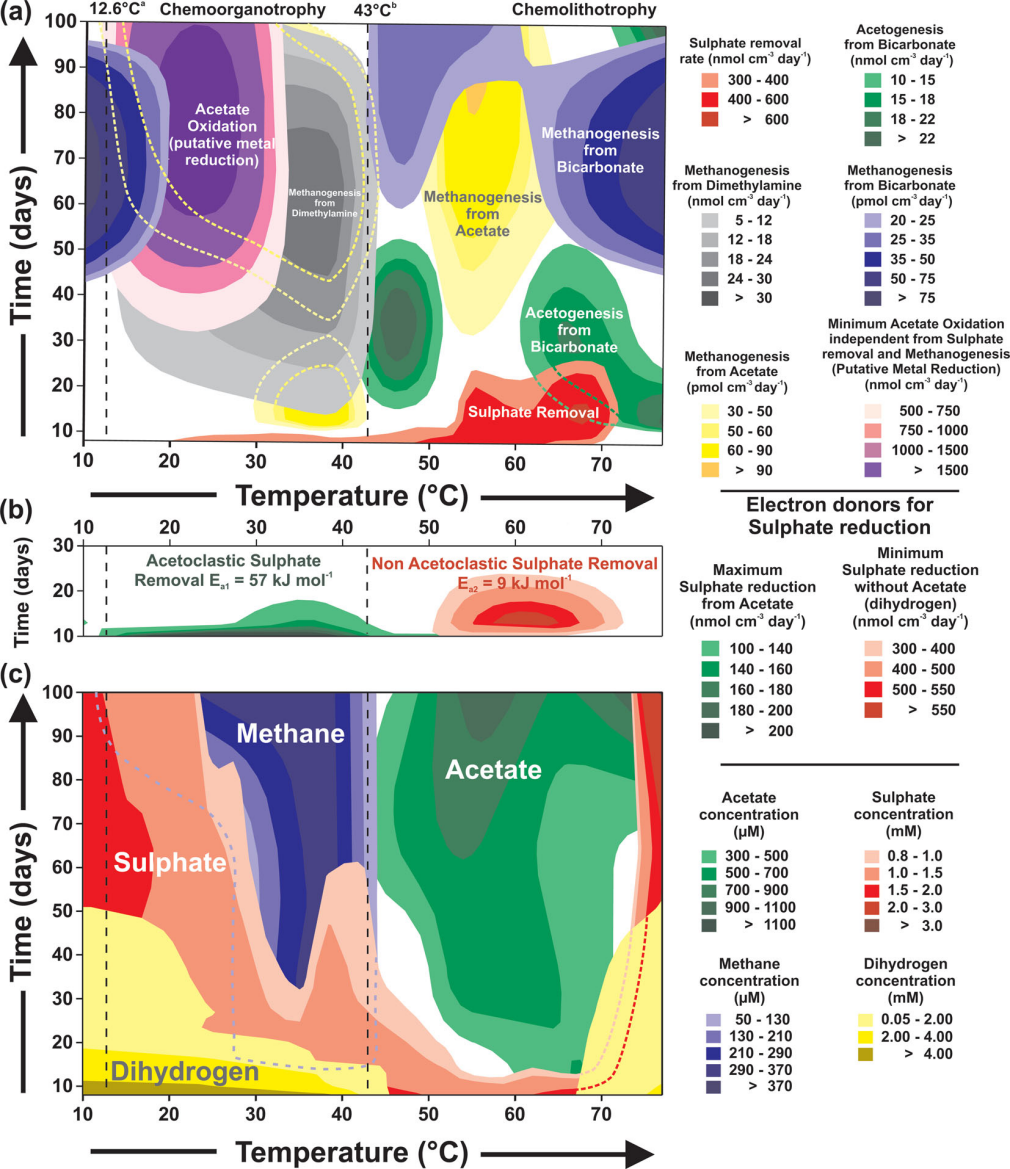
Effect of temperature and incubation time on sediment slurries (∼45 μM H_2_). (**a**) Specific metabolic activities (very low metabolic rates are not shown). Yellow dashed lines show acetoclastic methanogenic rates when overlaid by other activities. Green dashed lines show rates of autotrophic acetogenesis. (**b**) Putative substrates for sulphate reduction. (**c**) Main metabolic substrates and products. Blue dashed lines represent methane concentrations when overlaid by other compounds. Red dashed lines represent sulphate concentrations. ^a^Annual average *in situ* temperature. ^b^Temperature at which at least 50% of sulphate reduction was hydrogenotrophic.

Geochemical analysis of Portishead sediment cores showed that a range of anaerobic prokaryotic activities were cooccurring *in situ*: metal oxide and sulphate reduction; methanogenesis; acetate, H_2_ and ammonia formation (Fig. S1, Supporting Information). Interestingly, acetate and H_2_ concentrations were overall slightly higher in summer (19°C) than winter (7°C) (summer average acetate and H_2_ concentrations were 9 and 7 μM higher, respectively), which suggests increased organic matter hydrolysis/fermentation at the higher summer temperatures. This is consistent with organic matter reactivity increasing with increasing temperatures (Parkes *et al.* 2007b; Burdige 2011), but different to the experimental results of Weston and Joye (2005) who suggested that low molecular weight dissolved organic carbon should accumulate at low temperatures. Acetate concentrations also increased over time in the unamended sediment slurries at higher temperatures, above ∼40°C (Fig. 1). Below ∼40°C, however, there was a clear sequence of activities that occurred with increasing temperatures and time as indicated by geochemical changes (Fig. 1). From ∼10°C, both sulphate and H_2_ were slowly removed, and additionally above ∼23°C up to ∼45°C CH_4_ began to accumulate. Also at ∼45°C both H_2_ and sulphate became rapidly depleted and acetate accumulation began. Above ∼70°C, both H_2_ and sulphate removal slowed and eventually stopped with increasing temperature (Fig. 1). Acetate accumulation of >1 mM occurred above 50°C, as has been shown previously (Parkes *et al.* 2014).

Radiotracer activity measurements, however, demonstrated that changes in prokaryotic activity with temperature were more complex than reflected by geochemical changes, there being additional zones of different bacterial activities and changes in substrate utilization (Fig. 1). After about 40 days and at temperatures up to ∼20°C, hydrogenotrophic methanogenesis occurred. Although this result differs from the finding of acetoclastic methanogenesis being dominant at low temperatures in rice field soils (Fey and Conrad 2000), it is consistent with the presence of hydrogenotrophic methanogenesis in some low temperature, near-surface, marine sediments (e.g. Parkes *et al.* 2007a; Webster *et al.* 2009). This difference between rice field soils and marine sediments may reflect that generally freshwater systems tend to be dominated by acetoclastic methanogenesis, whilst marine sediments are dominated by hydrogenotrophic methanogenesis, as anaerobic metabolism is thought to be more focused on H_2_ as an intermediate (Whiticar 1999). The stimulation of hydrogenotrophic methanogenesis occurred in the presence of significant sulphate concentrations, which may be a reflection of acetate being the main substrate for sulphate reduction at low temperatures (Fig. 1, and subsequent discussion), and hence, not competing for H_2_.

Above about 12°C, with increasing temperatures, first acetate oxidation and then increasingly sulphate removal, and methylotrophic and acetoclastic methanogenesis became significant, followed above ∼30°C by an additional discrete zone of acetoclastic methanogenesis for up to ∼30 days (Fig. 1). Above 43°C there was a marked change in processes, with a clear decrease in CH_4_ formation and an increase in acetate concentrations. Below 43°C the maximum rates of acetate oxidation to carbon dioxide exceeded sulphate reduction rates (≤122 times) and also occurred ∼50 days after the sulphate reduction rates had peaked; hence, total acetate oxidation must have involved an electron acceptor in addition to sulphate (Fig. 1). As this ‘additional’ acetate oxidation was also 3 × 10^4^ times higher than the maximum rate of acetoclastic methanogenesis, this process could not have been responsible for the large ‘non-sulphate reduction’ acetate oxidation. It seems most likely that the use of metal oxides (e.g. manganese or iron oxides) for acetate oxidation was responsible for this ‘non-sulphate reduction, acetate-oxidation’, especially as other electron acceptors were limited in these anoxic sediments (e.g. nitrate concentrations <1 μM; Webster *et al.* 2010). Moreover, the *in situ* presence of dissolved pore water manganese and iron (Fig. S1, Supporting Information), significant concentrations of sedimentary metal oxides (iron and manganese oxides 5.3 and 0.1% of dry sediment, respectively) and phylogenetic analysis of active communities (Webster *et al.* 2010), all indicate that chemoorganotrophic metal reduction probably represents a significant process in these sediments. Hence, in agreement with *in situ* results for other coastal sediments (Canfield *et al.* 1993; Finke *et al.* 2007), in these slurries, sulphate reduction and putative metal oxide reduction likely accounted for most of the organic matter mineralization (26.5 and 73.2% respectively), despite the heating and H_2_ addition (Fig. 1).

**Figure 2.**
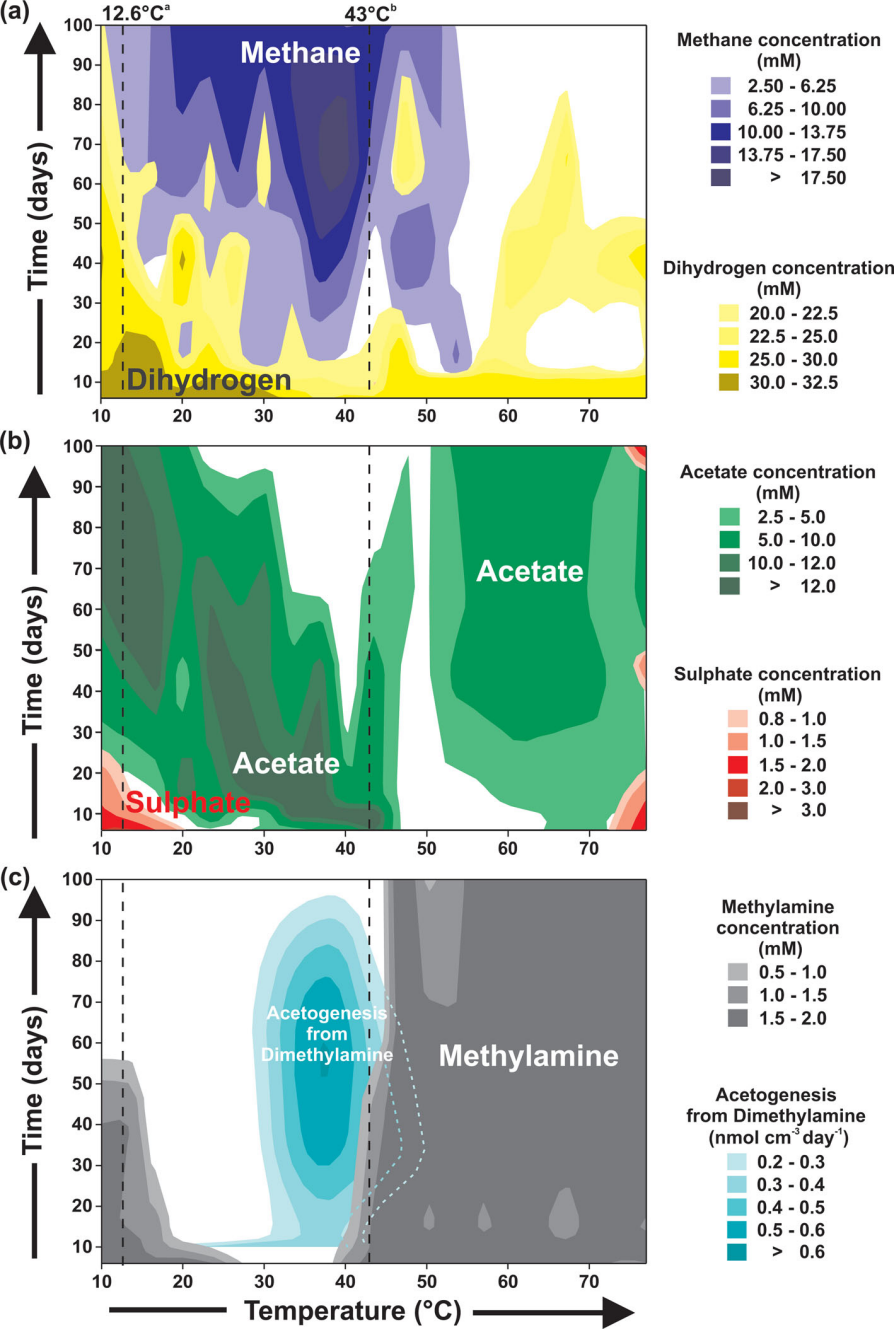
Effect of temperature and incubation time on the main substrate and product concentrations in substrate-amended sediment slurries (acetate, methylamine, H_2_). (**a**) Methane and H_2_ concentrations. (**b**) Acetate and sulphate concentrations. (**c**) Methylamine concentrations (between 10 and 100 days) and rates of methylotrophic acetogenesis in substrate-unamended slurries (∼45 μM H_2_). ^a^Annual average *in situ* temperature. ^b^Temperature at which at least 50% of sulphate reduction could have been hydrogenotrophic.

The occurrence of methylotrophic methanogenesis above 12°C, even in the presence of significant sulphate concentrations (Fig. 1), is understandable because methylamines are not direct substrates for sulphate-reducing bacteria, and these ‘non-competitive’ substrates preferentially stimulate methanogenesis (Oremland, Marsh and Polcin 1982). Similarly, methylotrophic methanogenesis in the sediment *in situ* could explain the presence of CH_4_ alongside high sulphate concentrations (>15 mM, Fig. S1, Supporting Information). However, methylated amine concentrations were always low in Portishead sediments (below ∼120 μM detection limits), implying a rapid turnover *in situ* controlled by the initial depolymerization/hydrolysis of organic matter (Arnosti 2004; Parkes *et al.* 2012). Significant rates of methylotrophic methanogenesis above ∼12°C in the slurries, therefore, suggests that the supply of methylated substrates was increased by heating, making precursor substrates more bioavailable (Burdige 2011). This increased bioavailability may also have been responsible for the stimulation of methylotrophic acetogenesis (Fig. 2) and acetoclastic methanogenesis above ∼30°C (Fig. 1). Only the high rates of methylotrophic methanogenesis coincided with considerable CH_4_ accumulation (Fig. 1); this suggests that some of the CH_4_ produced from low rates of hydrogenotrophic methanogenesis at lower temperatures was anaerobically oxidized in the slurries (ANME 2a and 2b capable of anaerobic oxidation of methane were detected). This situation also occurs *in situ*, for example, in the coastal sediments of the Danish Skagerrak; there were low levels of both hydrogenotrophic methanogenesis and anaerobic oxidation of methane in the shallow subsurface, and no CH_4_ was present (Parkes *et al.* 2007a).

Abruptly, above 43°C, there was almost no methylotrophic methanogenesis and no CH_4_ accumulation (Fig. 1). In addition, methylotrophic acetogenesis slowed dramatically (Fig. 2). However, sulphate reduction continued and at faster rates, and first autotrophic acetogenesis developed and then, after about 60 days, this was replaced by hydrogenotrophic methanogenesis (Fig. 1). Also, H_2_ was removed to below detection limits. Between 37 and 75°C, sulphate reduction rates exceeded total acetate oxidation rates (Fig. S2a, Supporting Information). As this coincides with the zone of decreasing and zero H_2_ concentrations (Fig. 1), this strongly suggests that H_2_ became a major substrate for sulphate reduction at higher temperatures. Calculations indicate that at 60°C, at least 71% of sulphate reduction occurred without acetate as a substrate (Fig. S2a, Supporting Information). Interestingly, at 43°C at least 50% of sulphate reduction was using H_2_ as a substrate, although below this temperature, acetate was the main sulphate reduction substrate (Fig. S2 c, d and f, Supporting Information). Therefore, 43°C is also an important critical temperature for sulphate reduction, marking a switch from predominantly organotrophic to lithotrophic metabolism. Sulphate was only completely removed at temperatures where H_2_ was the main substrate for sulphate reduction (Fig. 1). This was probably due to organic substrate limitation because in the replicate substrate-amended slurries (2 mM acetate and methylamine), in addition to H_2_ (∼36 mM gas equivalent), sulphate was completely removed between 3.2 and 73.8°C (Fig. 2b).

As sulphate removal rates increased to maximal above ∼53°C, abruptly autotrophic acetogenesis ceased, and when sulphate was depleted, a zone of acetoclastic methanogenesis developed with maximum rates at ∼55°C and after 80 days (Fig. 1). This change in metabolism occurs at the same temperature, >50°C, as that associated with the switch to the dominance of syntrophic acetate oxidation coupled with hydrogenotrophic methanogenesis in other anaerobic environments (e.g. Ho, Jensen and Batstone 2013). Dolfing, Larter and Head (2008) suggested that there are thermodynamic ‘windows of opportunity’ for various anaerobic metabolisms involving methanogenesis, and the ‘window of opportunity’ in these marine sediment slurries above ∼50°C may favour acetoclastic methanogenesis rather than syntrophic acetate oxidation. The developing high acetate concentrations (∼1 mM and higher) combined with zero H_2_ concentrations may be aspects of this slurry, which provide a window for acetoclastic methanogenesis between about 50 and 65°C (Fig. 1). At higher temperatures sulphate removal still occurred, but in association with autotrophic acetogenesis again. By about 20 days, however, sulphate reduction became sulphate limited and autotrophic acetogenesis occurred on its own. This acetogenesis was replaced by hydrogenotrophic methanogenesis after about 50 days. The temporal sequence of both these H_2_ utilizing processes continued at increasing temperatures, although with an increasing time gap between them, as the zone for autotrophic acetogenesis shrank faster than the zone for hydrogenotrophic methanogenesis expanded (Fig. 1). Despite these active H_2_ utilizing processes at elevated temperatures, above ∼67°C, added H_2_ removal became restricted. However, it is unclear whether H_2_ consumption at these high temperatures became balanced by H_2_ formation from sedimentary organic matter (Parkes *et al.* 2007b). The upper temperature limit for sulphate reduction in these slurries was ∼73°C (Fig. 1). This upper temperature is almost identical to that of a thermophilic, spore-forming, sulphate-reducing bacteria (*Desulfotomaculum* sp. C1A60, phylum *Firmicutes*) previously isolated from Portishead sediments, when growing on H_2_ (72°C, O'Sullivan *et al.* 2015). Thermophilic, spore-forming sulphate-reducing bacteria are widespread in marine sediments (Muller *et al.* 2014). As the highest acetate concentrations occur separately from the main zones of autotrophic acetogenesis (Fig. 1), an additional source of acetate formation must occur, which is presumably, heterotrophic, associated with the temperature activation of organic matter (Parkes *et al.* 2007b).

### The effect of different temperatures on anaerobic metabolism in substrate-amended sediment slurries

Similar results to the above also occurred in the substrate-amended slurries, with the higher substrate concentrations allowing their utilization to be directly analysed (Fig. 2). The total sulphate reduction temperature range remained unchanged with substrate addition (<3 to 73°C), but rates were faster and sulphate depletion more extensive. These results suggest that the syntrophs supplying sulphate-reducing bacteria with substrates may have the same temperature range as the sulphate-reducing bacteria, or that sulphate-reducing bacteria were independent of syntrophs, which seems unlikely as close coupling between fermenters and sulphate reducers has been previously shown (Finke and Jørgensen 2008). In contrast, addition of substrates did extend the temperature range for CH_4_ formation, especially at lower temperatures, from 23–44°C to 7–55°C. Hence, methanogens had a wider temperature range than their syntrophs and appeared to be substrate limited in the unamended slurries at temperatures below ∼20°C. However, curiously, in both slurry conditions the highest H_2_ concentrations occurred below ∼30°C (Figs 1 and 2), and in the non-substrate-amended slurries there was also H_2_ formation at these temperatures (>45 μM) and stimulation of hydrogenotrophic methanogenesis. Perhaps at these temperatures and conditions, there is uncoupling between H_2_ formation and consumption, H_2_ leakage (Finke *et al.* 2007) or other limitations on CH_4_ production.

Methylamine degradation did occur at low temperatures, but was much slower below than above the average *in* situ sediment temperature (12.6°C, Fig. 2) and was associated with acetate formation. For example, complete methylamine removal took ∼55 days at ∼10°C, compared to a few days above 20°C. As maximum methylamine degradation did not occur around the average *in situ* temperatures, other factors than just temperature adaptation must control optimal methylamine degradation. Methylamine degradation also resulted in ammonium accumulation, with maximum concentrations being reached between 10 and 15 days and at 20–40°C (4 mM). However, with continued incubation maximum ammonium concentrations decreased (reaching a minimum of ∼2 mM between 20 and 28°C), suggesting that anaerobic ammonium oxidation was occurring. Above 43°C, methylamine degradation stopped abruptly. This was consistent with the cessation of ^14^C-dimethylamine metabolism (methanogenesis and acetogenesis) in the non-substrate-amended slurries (Figs 1 and 2). Also similar to the non-substrate-amended slurries, acetate accumulated above 43°C, but concentrations were overall lower than below 43°C, in part probably due to methylamine degradation to acetate below 43°C. Complete H_2_ removal over time above ∼43°C broadly reflected the two hydrogenotrophic methanogenic zones in the non-substrate-amended slurries (Fig. 1). Despite this indication of hydrogenotrophic methanogenesis above ∼55°C and its direct measurement in non-substrate-amended slurries, no cooccurring CH_4_ was present in either slurry. This might be due to CH_4_ oxidation at higher temperatures, above ∼45°C (Kallmeyer and Boetius 2004; Holler *et al.* 2011).

### The effect of different temperatures on bacterial and archaeal cell numbers in sediment slurries

**Figure 3.**
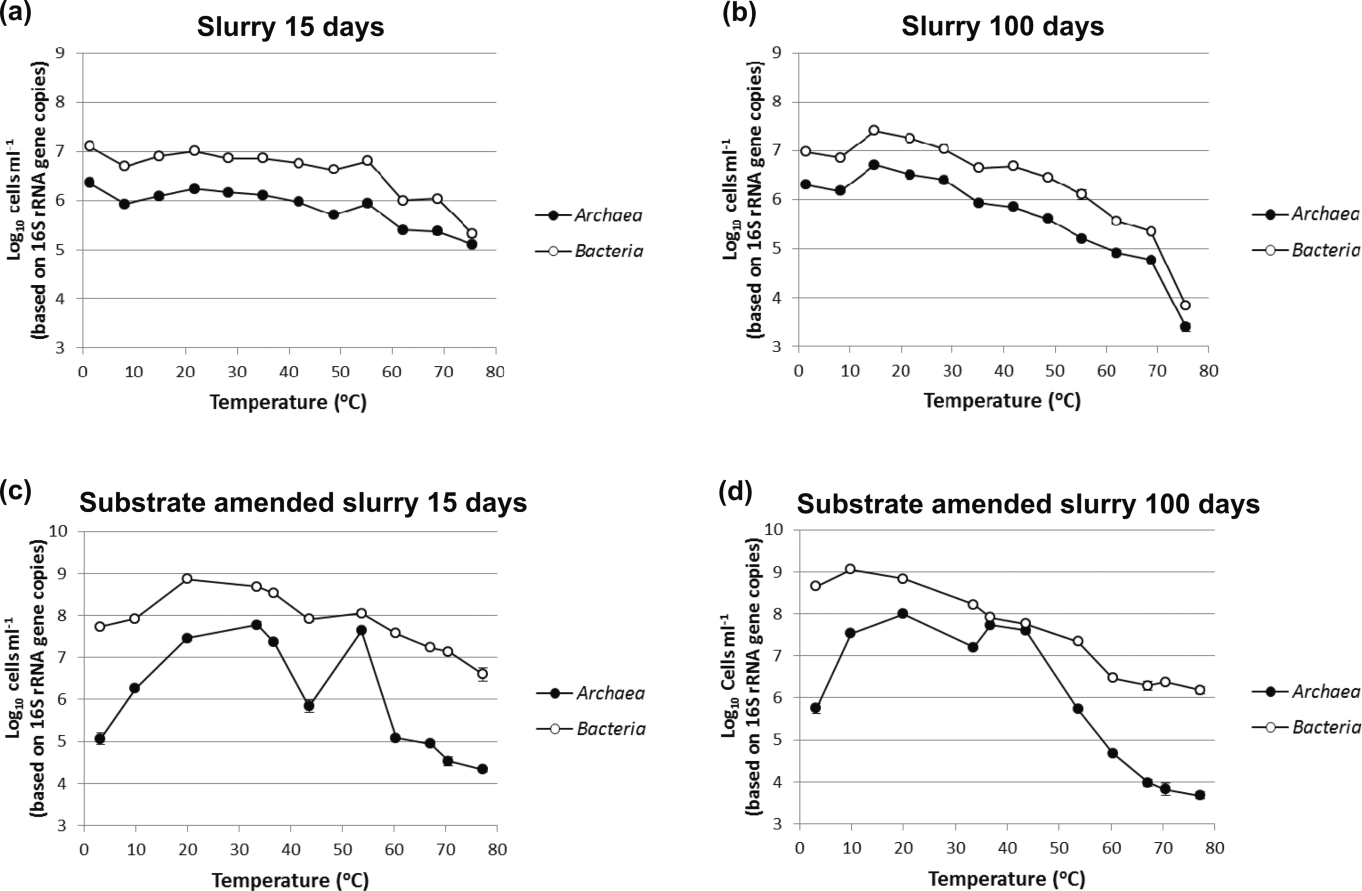
The effect of temperature and time (15 and 100 days) on *Bacteria* and *Archaea* cell numbers (16S rRNA gene copies) in unamended (**a** and **b**) and substrate amended (**c** and **d**) sediment slurries. Cell numbers were calculated from 16S rRNA gene copy numbers by using the average 16S rRNA gene copy number for each taxa (4.19 and 1.71 copies for *Bacteria* and *Archaea*, respectively) deduced using the rrnDB database (Stoddard *et al.* 2015). Standard deviations are plotted but are mostly within the size of the symbols.

Considerable numbers of bacterial and archaeal cells were in the non-substrate-amended slurries at 15 days (∼10^6^–10^7^ cells based on 16S rRNA gene copies mL^−1,^ Fig. 3), with bacterial cell numbers being consistently higher than archaeal cell numbers. Both archaeal and bacterial cell numbers decreased markedly above about 55°C. After 100 days, bacterial and archaeal cell numbers had increased with a peak at ∼15°C, slightly above the annual average *in situ* temperature (12.6°C), and close to the zone of maximum acetate oxidation (Fig. 1). Cell numbers subsequently decreased with increasing temperature, for *Bacteria*, in a bimodal pattern with relatively low numbers at ∼35°C (∼4.5 × 10^6^ ml^−1^), and overall, decreasing slightly faster than archaeal cells. Above 70°C for both bacterial and archaeal cells, there was a more dramatic decrease. These differences between bacterial and archaeal cell distributions between ∼15 and 70°C perhaps may be due to a combination of the inherently greater thermal tolerance of archaeal membranes (Koga 2012) and germination of thermophilic bacterial spores at higher temperatures (corresponding with an intense zone of thermophilic sulphate reduction [Fig. 1]). However, for both cell types the 100-day cell numbers were lower above ∼50°C compared to the 15 day counts; hence, prokaryotic populations overall decreased due to prolonged incubation at thermophilic temperatures.

**Figure 4.**
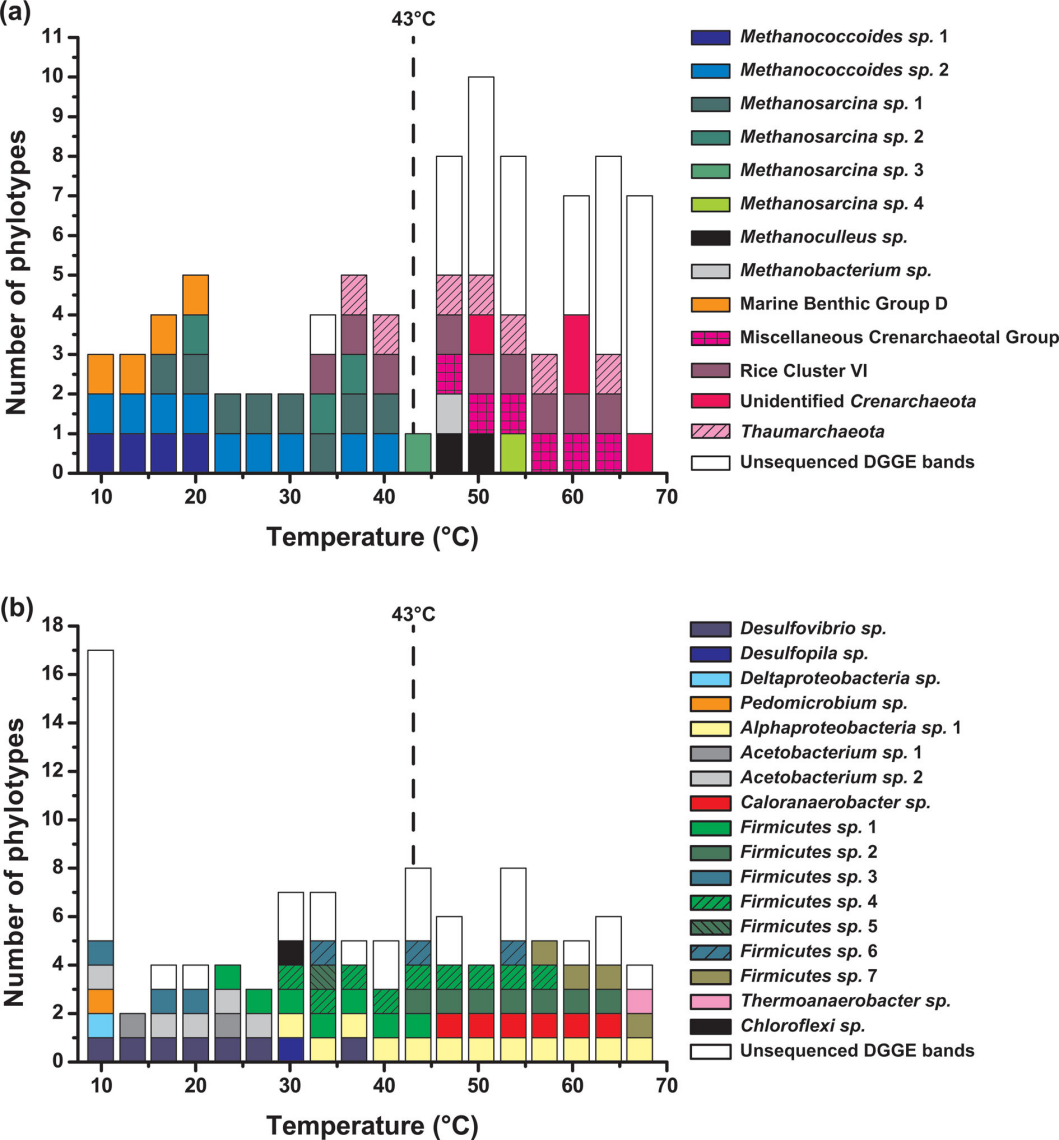
The effect of temperature on 16S rRNA gene diversity (PCR-DGGE) in substrate-amended slurries incubated for 100 days at different temperatures. (**a**) *Archaea* and (**b**) *Bacteria*.

The cell distributions with temperature in the substrate-amended slurries were similar to the above for *Bacteria*, except numbers were over a factor of 10 higher and did not decrease so markedly with temperature, especially above 60°C (Fig. 3). This difference may reflect that bacterial populations with adequate substrate supply were able to respond more effectively to increasing temperatures, such as by membrane lipid changes, and greater spore germination/growth of thermophiles [e.g. increased detection of *Firmicutes* (Fig. 4) and more complete sulphate removal (Fig. 2)]. Archaeal cell distributions in the substrate-amended slurries were very different, with two peaks: a broad peak around ∼20 to 30°C and a sharper peak at ∼40 to 50°C, present at both 15 and 100 days. This also indicates growth of archaeal cells, although restricted to around meso and lower thermophilic temperatures. Surprisingly, at 100 days archaeal cell numbers decreased more rapidly at temperatures above ∼43°C than did bacterial cells, which is opposite to what occurred in the non-substrate added slurries. This decrease in archaeal cells coincides with the cessation in methylamine degradation (Figs 1 and 2), suggesting that some *Archaea* may have a role in anaerobic degradation of methylamines, and is another impact of the 43°C critical temperature. In addition, above ∼57°C archaeal cell numbers in the substrate-amended slurry at both 15 and 100 (except 76°C at 100 days) days were actually lower than in the non-substrate-amended slurry. These data suggest that at elevated temperatures, the presence of significant substrate concentrations is detrimental to many archaeal cells, whilst stimulating bacterial populations (Fig. 3). Surprisingly, bacteria dominate the total prokaryotic population at all temperatures, at both 15 and 100 days, and with and without added organic substrates. The theoretically more thermally robust archaeal cells (Valentine 2007; Koga 2012) were thought to be dominant in subsurface sediments (Biddle *et al.* 2006; Lipp *et al.* 2008) and some high-temperature environments, but here they only became significant around the two peaks in their cell numbers at ∼30 and 50°C (10 and 40%, respectively of the total population).

### The effect of different temperatures on prokaryotic community composition in sediment slurries

Cluster analysis of PCR-DGGE (Fig. S3, Supporting Information) and ARISA data (not shown) of the unamended slurries showed a distinct bacterial community change at temperatures above 40°C already at 15 days. These temperature effects were expanded by 100 days with distinct bacterial communities at 8–22°C, 25–35°C, 39°C, 42–62°C and 69–75°C (Fig. S3, Supporting Information), and for *Archaea* at 5°C, 8–15°C, 18–45°C, 49°C, 39–65, 52–59°C, 62°C, 69–72°C and 75–79°C (Fig. S4, Supporting Information). Overall, at both 15 and 100 days there was a decrease in the number of phylotypes above ∼40°C. In addition, the 100-day slurry incubated at 1°C clustered with the original slurry, indicating that low temperature incubation in the Thermal Gradient did not change the original slurry community and observed changes were, therefore, a result of the elevated incubation temperatures. The prokaryotic composition of the original slurry was also similar to the fresh sediment (based on 454 pyrosequencing of 16S rRNA genes, unpublished results), so the Thermal Gradient community changes should be representative of the response of the original sediment community. The large number of faint DGGE bands in the unamended slurries made excision and sequencing of bands difficult; however, successful sequencing of some intensely stained bands for *Bacteria* showed the presence of *Deltaproteobacteria* below ∼30°C, *Deltaproteobacteria* and *Firmicutes* between ∼30 and 35°C and then a mixture of different *Firmicutes*, including *Clostridia*-like *Bacteria*, above ∼40°C.

The presence of *Deltaproteobacteria* and *Firmicutes* was also confirmed by pyrosequencing of 16S rRNA gene amplicons and these together with *Chloroflexi*, *Actinobacteria*, *Bacteroidetes*, *Acidobacteria* and candidate division OP8 were the most abundant bacterial phyla (90%; Fig. S5, Supporting Information). *Proteobacteria*, including sequences related to the psychrophilic and heterotrophic sulphate-reducing bacteria, *Desulfotalea* spp. and *Chloroflexi* were most abundant at low temperatures, but decreased with increasing temperature significantly as *Firmicutes*, including sequences related to thermophilic, spore-forming and H_2_-utilizing sulphate-reducing bacteria, *Desulfotomaculum* spp. (Hubert *et al.* 2010; de Rezende *et al.* 2013; O'Sullivan *et al.* 2015) proliferated toward a peak abundance at 46°C (80% of bacterial phylotypes). *Acidobacteria*, *Bacteriodetes* and candidate division OP8 also decreased with increasing temperature, whereas *Actinobacteria* slightly increased (Fig. S5, Supporting Information). Members of the phylum *‘Bathyarchaeota’ (formerly MCG;* Meng *et al.* 2014) were the most abundant archaeal phylum at all times and temperatures based on pyrosequencing (consistently >45% of *Archaea*). However, different bathyarchaeotal phylotypes were abundant at different temperatures suggesting considerable physiological diversity within the group. *Thaumarchaeota* (putative ammonium oxidizers), *Euryarchaeota* [including the H_2_-utilizing methanogenic *Methanomassiliicocaceae* (Dridi *et al.* 2012); *Methanobacteriales* and *Methanomicrobiales;* the substrate versatile methanogens *Methanosarcinales*, which were most abundant*;* and sequences related to the anaerobic methane oxidising clade ANME 2a-2b, most abundant at 35°C] and *Parvarchaeota* (Rinke *et al.* 2013) were also present but in much lower proportions (Fig. S6, Supporting Information).

Consistent with the significant growth in both bacterial and archaeal populations at a range of temperatures (Fig. 3), PCR-DGGE profiles of the substrate-amended slurries had more intensely stained bands compared to those of the unamended slurries, and this allowed robust sequence analysis. These sequences presumably represent prokaryotes that had grown under their optimum geochemical and temperature conditions, and predominated over organisms just surviving or dying slowly; hence, the community composition of the substrate-amended slurries may provide a clearer link to the changes in metabolism at different temperatures. However, there were still considerable similarities between the community composition changes with temperature in both slurry conditions. In the substrate-amended slurries below ∼20°C where there was both active sulphate reduction and methanogenesis, but neither sulphate or H_2_ was depleted (Fig. 2), the bacterial community was dominated by *Deltaproteobacteria*, including organotrophic, incomplete oxidizing sulphate-reducing bacteria (*Desulfovibrio*), *Acetobacterium* (acetogens) and different *Firmicutes* (Fig. 4), consistent with sulphate removal and acetate formation. The *Archaea* were dominated by the methylotrophic *Methanococcoides* methanogens (Fig. 4), presumably responsible for the limited non-competitive methylamine utilization, and hence, CH_4_ production in the presence of sulphate. Marine Benthic Group D/*Thermoplasmatales* sequences were also present and although these are presently uncultured, genome data indicates that they are capable of exogenous protein degradation in cold anoxic environments (Lloyd *et al.* 2013), whilst phylogenetic analysis suggests that some clades may even be methanogenic (Paul *et al.* 2012). Their presence, however, was restricted to temperatures below ∼20°C. Around 20°C substrate versatile *Methanosarcina* methanogens appeared, in addition to *Methanococcoides*, and were present up to the 43°C critical temperature, where they became the sole archaeal phylotype present. This temperature range corresponds with maximum CH_4_ concentrations and methylamine utilization (Fig. 2). Above ∼20°C the bacterial community also changed with additional types of *Firmicutes* present, this corresponded with more rapid methylamine and sulphate removal, and acetate production (Fig. 2). Above 30°C *Alphaproteobacteria* appear along with different *Firmicutes*. *Alphaproteobacteria* were then consistently present up to 70°C, members of this highly diverse group have been detected in a subsurface oil reservoir (Kryachko *et al.* 2012) and hydrothermal vents (Takai *et al.* 2009).

At 43°C the composition of *Firmicutes* again changed (*Firmicutes* sp. 2 appear, Fig. 4), and at slightly higher temperatures this was augmented by *Caloranaerobacter*, a thermophilic, anaerobic, organotrophic bacterium, species of which have been isolated from deep-sea hydrothermal vents (Wery *et al.* 2001; Jiang *et al.* 2014), and which can produce acetate as a fermentation product. *Caloranaerobacter* was present up to ∼65°C, which is the upper temperature limit for cultured isolates (Wery *et al.* 2001), and acetate concentrations again increased (Fig. 2). Above ∼55°C other *Firmicutes* groups appeared (*Firmicutes* sp. 7, Fig. 4), including above ∼65°C *Thermoanaerobacter*, a heterotrophic, thermophilic anaerobe, the metabolism of which involves both acetate and H_2_, and some species have been isolated from the deep subsurface (Fardeau *et al.* 2000; Roh *et al.* 2002). The composition of *Archaea* changes even more markedly above 43°C than did *Bacteria* (Fig. 4), from the single *Methanosarcina* phylotype to the first appearance of *Methanoculleus* and then its presence up to ∼50°C, which is consistent with a temperature window for hydrogenotrophic methanogenesis documented in the unamended slurries (Fig. 1) and continued CH_4_ formation in the amended slurries (Fig. 2). This upper temperature limit is the same as for *Methanoculleus submarinus* isolated from deep-sea sediments (247 m; Mikucki *et al.* 2003). The hydrogenotrophic methanogen, *Methanobacterium* was also present but only at ∼45°C. This genus has thermophilic species (Zeikus and Wolfe 1972) and has also been detected in subsurface environments (Kotelnikova and Pedersen 1997; Kryachko *et al.* 2012). In addition, *Thaumarchaeota* originally first present above 35°C became a consistent member of the archaeal population (Fig. 4). All known members of *Thaumarchaeota* are chemolithoautotrophic ammonia oxidizers and this is consistent with ammonium removal between ∼20 and 40°C. However, they have not so far been shown to be capable of anaerobic growth (Stahl and de la Torre 2012) despite being common in anoxic, subseafloor sediments (Parkes *et al.* 2014). Our results suggest that some *Thaumarchaeota* may be capable of anaerobic metabolism. In addition, the continued presence of *Thaumarchaeota* above 40°C indicates metabolism other than ammonia oxidization, as suggested in some other environments (Mussmann *et al.* 2011; Stahl and de la Torre 2012). Above 45°C the common subseafloor ‘*Bathyarchaeota**’***/MCG appears and is present up to ∼65°C. These *Archaea* have been detected in sediments up to 95°C (Biddle *et al.* 2012) and are thought to be heterotrophic (Lloyd *et al.* 2013), and perhaps activation of recalcitrant organic matter at higher temperatures stimulated their growth (Parkes *et al.* 2007b). The temperature distribution of the *Bathyarchaeota/*MCG was similar to another uncultured archaeal group, the probably non-methanogenic Rice cluster VI (Fey and Conrad 2000) which has also been found in deep, thermophilic, subseafloor sediments (Roussel *et al.* 2008).

These biodiversity results demonstrate that there are clear changes in community composition with temperature, often over narrow temperature intervals, and that these changes match those in biogeochemical activity, and the temperature ranges of cultivated representatives. The distribution and activity of methanogens, sulphate-reducing and acetogenic bacteria was supported by the distribution of their functional genes (*mcrA*, *dsrB* and *fths*, respectively, unpublished results).

### The dominant anaerobic metabolic processes in substrate-unamended slurries

**Figure 5.**
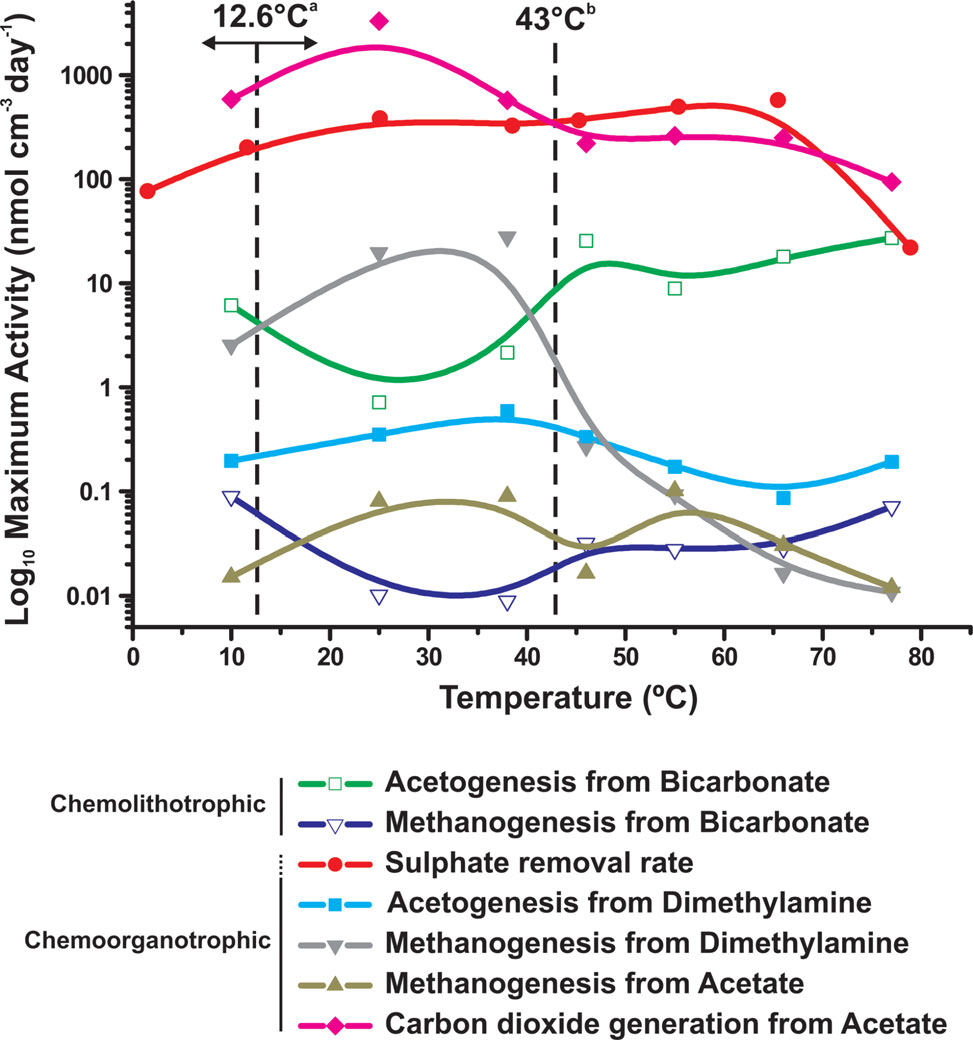
Maximum activity rates for studied metabolic processes at different temperatures in sediment slurries (H_2_ ∼45 μM). ^a^Annual average *in situ* temperature (Arrows indicate range between minimum and maximum *in situ* temperature). ^b^Temperature at which at least 50% of sulphate removal was hydrogenotrophic.

Regardless of the temperature, sulphate reduction and acetate oxidation were major metabolic activities in these coastal sediment slurries (Fig. 5), with highest activity rates being carbon dioxide from acetate oxidation (1644 nmol cm^−3^ day^−1^ at 25°C) and sulphate reduction (602 nmol cm^−3^ day^−1^ at 69°C) (Table S2, Supporting Information). Acetate was an important compound over the whole temperature range (10–77°C), both as a substrate for some metabolic processes and product of others. Total acetate oxidation to carbon dioxide increased with temperature up to a maximum at 25°C, with rates 7.5 times greater below 40°C than above this temperature. As previously described, metal oxide reduction must have been responsible for the majority of acetate oxidation, as often occurs in coastal marine sediments (Canfield *et al.* 1993).

The effect of temperature on sulphate reduction rates was also variable with a bimodal peak (*R*^2^ = 0.78, Fig. 1 and S2, Supporting Information), suggesting that total sulphate removal was due to two sulphate reduction processes with different optimal temperatures (29 and 61°C), as found in other sediments (Isaksen, Bak and Jørgensen 1994; Hubert *et al.* 2009). Here we also show that the two sulphate reduction processes were probably utilizing different substrates (Fig. 1b), and likely involved different bacteria (Fig. 4). Sulphate reduction was the second most important chemoorganotrophic process (Fig. 5) and below 43°C was most likely coupled to acetate oxidation. That this activity was due to substrates other than acetate (e.g. lactate, propionate, butyrate and valerate; Parkes *et al.* 1989) was unlikely, as these compounds were either below detection limit (<1 μM) or their concentration profiles were not correlated with sulphate reduction (data not shown). Acetate is also the major *in situ* substrate for sulphate reduction in marine sediments (Parkes *et al.* 1989). Between 37 and 75°C, however, sulphate reduction rates exceeded total acetate oxidation rates (Fig. S2, Supporting Information) and coincided with complete removal of H_2_ (Fig. 1), which strongly suggests that H_2_ had become the major substrate for sulphate reduction (e.g. 71% of sulphate reduction at 60°C, Fig. S2, Supporting Information). Interestingly, at the 43°C critical temperature H_2_ was probably driving at least 50% of sulphate reduction, with acetate responsible for the rest (Fig. S2, Supporting Information).

A switch from acetoclastic to hydrogenotrophic anaerobic terminal oxidizing processes has been reported previously but for methanogenesis in rice fields and lake sediments at temperatures higher than about 30–40°C (Fey and Conrad 2000; Nozhevnikova *et al.* 2007). The temperature-driven transition between substrates for sulphate reduction in these slurries might be a consequence of (1) acetoclastic sulphate and metal oxide reducers being restricted by temperature, as suggested by the change in bacterial diversity (Fig. 4); (2) acetoclastic processes being outcompeted for electron acceptors by hydrogenotrophic processes at high temperatures; and/or (3) specific syntrophic relationships between chemoorganotrophic acetogens and acetate oxidizers up to ∼40°C might become disrupted at higher temperatures, resulting in H_2_ becoming the dominant anaerobic intermediate and acetate accumulation (Fig. 1c). Assuming that this also occurs *in situ*, it would provide an additional mechanism for acetate accumulation in deep sediments above ∼40°C (Parkes *et al.* 2007b), including petroleum reservoirs (Seewald 2003).

### Carbon mineralization controls and temperature

**Figure 6.**
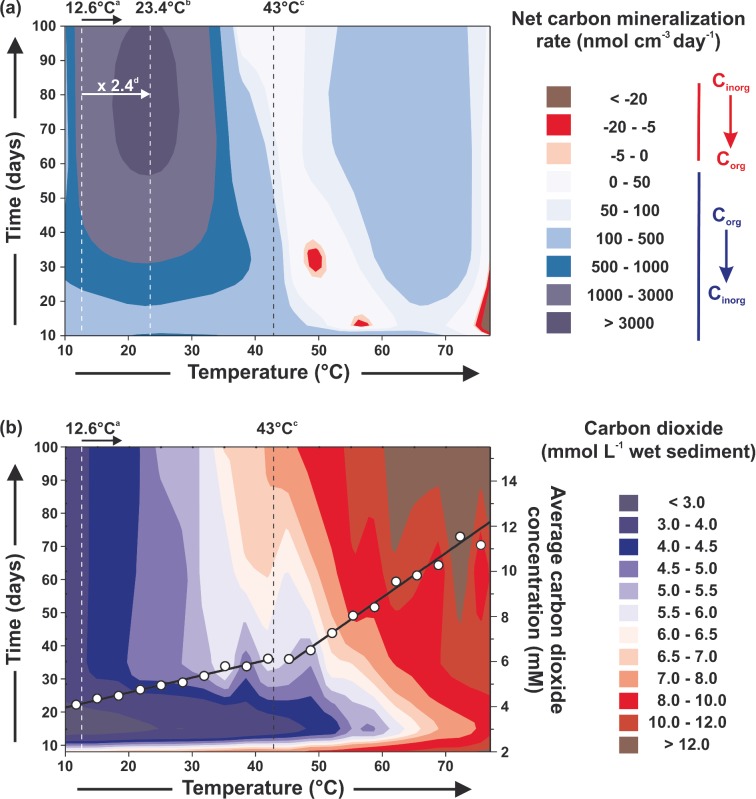
(**a**) Effect of temperature and incubation time on the net carbon mineralization balance of sediment slurries (∼45 μM H_2_). Net carbon mineralization balance was calculated by applying the standardizing factor in Table S1 (Supporting Information) to each specific rate, and then summing these. (**b**) Effect of temperature and incubation time on carbon dioxide concentrations measured from the slurry headspace (left vertical axis) and on the average carbon dioxide concentrations (open circles and right vertical axis). ^a^Annual average *in situ* temperature (Arrow indicates maximum average *in situ* temperature). ^b^Temperature of maximum mineralization rate. ^c^Temperature at which at least 50% of sulphate reduction was hydrogenotrophic. ^d^Factor of increase of mineralization rate.

Although the Portishead sediment depth profiles had very low sulphate reduction activity (Fig. S1, Supporting Information), sulphate removal rates in unamended Portishead sediment slurries (plus H_2_) were in the range for active coastal sediments (Jørgensen 1982), demonstrating that some anaerobic metabolic processes in these tidal flat sediments were strongly substrate limited. Anaerobic processes were also strongly controlled by temperature, as has been previously shown (e.g. Middelburg *et al.* 1996); however, changes were not linear and the response of individual processes to temperature increases was variable (Fig. 5). In addition, average net total carbon dioxide production rates (calculated from overall carbon dioxide production and consumption rates of each metabolic activity) were actually greater below 43°C (8.7 times, 781 nmol cm^−3^ day^−1^) than above this temperature. Hence, processes oxidizing organic carbon dominated below 43°C (Fig. 6). Marked changes in archaeal and bacterial diversity also occurred with temperature, particularly around this 43°C critical temperature (Fig. 4, S3–6, Supporting Information), demonstrating that distinct prokaryotic communities with specific metabolisms for each temperature ‘window of opportunity’ (Dolfing, Larter and Head 2008) were driving the temperature-related biogeochemical changes (Figs 1 and 2). Rates of chemoorganotrophic methanogenesis, acetogenesis, sulphate and putative metal oxide reduction all decreased rapidly above 43°C, whilst chemolithotrophic sulphate reduction, acetogenesis and methanogenesis were all stimulated (Figs 1 and 2), suggesting that a common factor might control the changes in both types of processes. Carbon dioxide partial pressures increased with temperature and time, particularly above 43°C where rates of increase were three times greater (Fig. 6b). Since biotic organic matter oxidation rates decreased above 43°C (Fig. 6a), this carbon dioxide increase must have been due to other mechanisms, such as decreased carbon dioxide solubility (Fig. S7, Supporting Information), and slurry acidification from organic acid accumulation (e.g. acetate; Fig. S7, Supporting Information). However, the main driving processes were probably related to the type of catagenic reactions occurring above ∼50°C during organic matter burial and maturation (Horsfield *et al.* 2006) responsible for increasing carbon dioxide concentrations in petroleum formations (Seewald 2003).

In these slurries (Fig. 6b), the increasing carbon dioxide concentrations with temperature may be responsible for enhancing chemolithotrophic metabolism, as increased carbon dioxide concentration has been shown to impact both prokaryotic activities and communities in other marine sediments (Mayumi *et al.* 2013; Yanagawa *et al.* 2013). In addition to the direct substrate increase for chemolithotrophic metabolism, carbon dioxide increase may cause product inhibition of organic carbon oxidation by chemoorganotrophic processes above ∼43°C. A potential causal relationship between the beginning of more rapid increases in carbon dioxide concentrations (Fig. 6b) at the 43°C critical temperature window and fundamental biogeochemical changes is suggested by the following: (1) the abrupt cessation of methylotrophic methanogenesis and acetogenesis, (2) the end of complete methylamine removal and (3) the reduction in biogenic carbon dioxide production, being combined with, (4) the start of hydrogenotrophic acetogenesis, (5) increasing dominance of hydrogenotrophic sulphate reduction, (6) prokaryotic diversity changes and (7) start of the rapid decrease in archaeal cell numbers in the substrate-amended slurries; all occurring around 43°C.

Different metabolic windows of opportunity are reflected in different optimal temperatures and incubation times for different anaerobic processes (Table S2, Supporting Information). Interestingly, the two autotrophic processes of acetogenesis and methanogenesis both had two temperature optima and these were the same (≤10°C, ≥77°C), whilst heterotrophic processes only had one temperature optimum and these varied from 25 to 55°C. Sulphate reduction, which had acetate as a substrate at lower temperatures but H_2_ as a substrate at higher temperatures (Fig. 1), had optimum activities during the zone of predominantly H_2_ utilization (∼95% H_2_ utilization, 69°C; Table S2 and Fig. S2, Supporting Information). Sulphate reduction also had two activation energies, the values during high-temperature H_2_ utilization being ∼6 times lower than during lower temperature acetate utilization (8.9 and 56.8 kJ mol^−1^), and hence, H_2_ utilization was a highly favoured reaction at higher temperatures which resulted in maximum activities and complete H_2_ removal (Fig. 1). The response of activities to a 10°C temperature increase (Q_10_ between 10 and 20°C, Table S2, Supporting Information) varied considerably from 1.1 (hydrogenotrophic sulphate reduction, acetoclastic methanogenesis) to 2.5 (hydrogenotrophic acetogenesis, methylotrophic methanogenesis), suggesting considerable differences in their response to climate warming and temperature increases during sediment burial.

## SUMMARY

It is generally considered that the impact of temperature on anaerobic biogeochemical processes in marine sediments, and other environments, would be controlled by the temperature characteristics of the prokaryotic community (e.g. Robador, Bruchert and Jørgensen 2009). Hence, there should be a progression from psychrophilic, mesophilic to thermophilic etc. populations for the dominant metabolism and generally increasing activities as temperatures increase. However, the results presented here, which greatly extend previous findings from a range of different environments (e.g. rice paddies, oil reservoirs, marine sediments, and unpublished mud volcano sediments studies), demonstrate that temperature increases have a direct, non-linear effect on the dominant biogeochemical processes and causative prokaryotes in a series of temperature windows of ‘opportunity’ with surprisingly rapid metabolic changes over small temperature increases (Figs 1 and 2). Here a critical 43°C temperature window was related to a more rapid increase in carbon dioxide concentrations (Fig. 6b) and this may also occur in other environments, particularly in subsurface sediments with increasing temperatures and carbon dioxide concentrations with depth (Seewald 2003). Deep hot sediments also have elevated acetate concentrations (Parkes *et al.* 2007b), as occurred in these slurries. Some activities such as hydrogenotrophic methanogenesis had multiple windows of opportunity within a large temperature range (∼10 to 80°C). Others including acetate oxidation had a more restrictive temperature range for maximum activities and this was also dependent on availability of electron acceptors [metal oxide (up to ∼34°C) and sulphate (up to ∼50°C), Fig. 1]. In addition, some activities switched substrates with temperature, this occurred for sulphate reduction, with acetate being the main substrate below 43°C and H_2_ being the main substrate above 43°C (Fig. 1, both with different energy of activations). This is consistent with (1) the widespread occurrence of spores of thermophilic sulphate-reducing bacteria, *Firmicutes—Desulfotomaculum* and other types, in near-surface, marine sediments (Muller *et al.* 2014) and their widespread activity in deep, hot sediments (Aullo *et al.* 2013) and (2) the prevalence of *Firmicutes* in our slurries and their species changes above 43°C (Fig. 4). A close relationship between functional biogeochemical changes and structural community changes occurred across the whole temperature range (∼0 to 80°C); for example, the presence of methylotrophic *Methanococcoides* methanogens was related to methylamine removal, methylotrophic methanogenic activity and methane formation (Fig. 1 and 2, and when methanogenic substrates changed above 43°C so did the types of methanogens present (Fig. 4). Several of the uncultured *Archaea*, characteristic of deep marine sediments, such as Marine Benthic Group D and Miscellaneous Crenarchaeatol Group/’*Bathyarchaeota’*, developed in the slurries and this indicated their possible metabolisms and temperature ranges.

Bacterial cell numbers were always higher than archaeal cells (Fig. 3), although in the non-substrate-amended slurries above ∼15°C bacterial cell numbers decreased rather more rapidly than the more robust archaeal cells with increasing temperatures. This situation has been suggested to occur in deep marine sediments (Schouten *et al.* 2010). Both bacterial and archaeal cell numbers decreased markedly above ∼70°C which could provide substrates in the form of necromass for prokaryotes able to grow at these temperatures (Parkes *et al.* 2014). Although both archaeal and bacterial cell numbers increased due to substrate addition, the pattern of their response was very different (Fig. 3). Archaeal cells increased around mesophilic and lower thermophilic temperatures (reaching ∼10 and 40% of the total prokaryotic population, respectively) and then decreased rapidly above the 43°C critical temperature. This coincided with the abrupt cessation in methylamine degradation, suggesting that some *Archaea* were degrading methylamines. Surprisingly, above ∼57°C archaeal cell numbers were actually lower than in the non-substrate-amended slurries (except 76°C at 100 days). This suggests that at these high temperatures, the presence of significant substrate concentrations is detrimental to some archaeal cells. In contrast, bacterial cell numbers were stimulated at all temperatures by substrate addition, including above 70°C (∼10 times higher). This differential response to substrate addition by *Bacteria* and *Archaea* with increasing temperatures may be significant in some high temperature and high substrate environments, such as, oil reservoirs.

In addition to temperature windows changing the structure and function of prokaryotic communities across a wide temperature range, the data presented here shows that even small temperature increases can have a significant impact on anaerobic organic matter degradation. For example, a temperature increase of only 2°C above the average annual *in situ* temperature of Portishead tidal flat sediments would increase organic carbon mineralization by 40% (Q_10_, 10–20°C, 1.1 to 2.5), further potentially contributing to total greenhouse gas emissions. Furthermore, the effect of temperature increase on organic carbon mineralization is also enhanced by substrate addition (Fig. 2). Hence, in addition to direct temperature effects of global warming, potential-associated eutrophication of coastal environments and elevated organic matter input would further increase the intensity of anaerobic activity and deleterious environmental impacts.

## Supplementary Material

Supplementary data are available at FEMSEC online.

## References

[bib1] Aller RC, Yingst JY (1980). Relationships between microbial distributions and the anaerobic decomposition of organic-matter in surface sediments of long-island sound, USA. Mar Biol.

[bib2] Altschul SF, Gish W, Miller W (1990). Basic local alignment search tool. J Mol Biol.

[bib3] Arnosti C (2004). Speed bumps and barricades in the carbon cycle: substrate structural effects on carbon cycling. Mar Chem.

[bib4] Aullo T, Ranchou-Peyruse A, Ollivier B (2013). Desulfotomaculum spp. and related Gram-positive sulfate-reducing bacteria in deep subsurface environments. Front Microbiol.

[bib5] Biddle JF, Cardman Z, Mendlovitz H (2012). Anaerobic oxidation of methane at different temperature regimes in Guaymas Basin hydrothermal sediments. ISME J.

[bib6] Biddle JF, Lipp JS, Lever MA (2006). Heterotrophic Archaea dominate sedimentary subsurface ecosystems off Peru. P Natl Acad Sci USA.

[bib7] Bragg L, Stone G, Imelfort M (2012). Fast, accurate error-correction of amplicon pyrosequences using Acacia. Nat Methods.

[bib8] Burdige DJ (2011). Temperature dependence of organic matter remineralization in deeply-buried marine sediments. Earth Planet Sc Lett.

[bib9] Canfield DE, Jørgensen BB, Fossing H (1993). Pathways of organic-carbon oxidation in three continental-margin sediments. Mar Geol.

[bib10] Caporaso JG, Kuczynski J, Stombaugh J (2010). QIIME allows analysis of high-throughput community sequencing data. Nat Methods.

[bib11] Conrad R, Klose M, Noll M (2009). Functional and structural response of the methanogenic microbial community in rice field soil to temperature change. Environ Microbiol.

[bib12] Conrad R, Wetter B (1990). Influence of temperature on energetics of hydrogen metabolism in homoacetogenic, methanogenic, and other anaerobic bacteria. Arch Microbiol.

[bib13] de Rezende JR, Kjeldsen KU, Hubert CRJ (2013). Dispersal of thermophilic Desulfotomaculum endospores into Baltic Sea sediments over thousands of years. ISME J.

[bib14] Delong EF (1992). Archaea in coastal marine environments. PNatl Acad Sci USA.

[bib15] DeSantis TZ, Hugenholtz P, Larsen N (2006). Greengenes, a chimera-checked 16S rRNA gene database and workbench compatible with ARB. Appl Environ Microb.

[bib16] Dolfing J, Larter SR, Head IM (2008). Thermodynamic constraints on methanogenic crude oil biodegradation. ISME J.

[bib17] Dridi B, Fardeau ML, Ollivier B (2012). Methanomassiliicoccus luminyensis gen. nov., sp. nov., a methanogenic archaeon isolated from human faeces. Int J Syst Evol Micr.

[bib18] Edgar RC (2010). Search and clustering orders of magnitude faster than BLAST. Bioinformatics.

[bib19] Fardeau ML, Magot M, Patel BKC (2000). Thermoanaerobacter subterraneus sp nov., a novel thermophile isolated from oilfield water. Int J Syst Evol Micr.

[bib20] Fey A, Conrad R (2000). Effect of temperature on carbon and electron flow and on the archaeal community in methanogenic rice field soil. Appl Environ Microb.

[bib21] Finke N, Hoehler T, Jørgensen B (2007). Hydrogen ‘leakage’ during methanogenesis from methanol and methylamine: implications for anaerobic carbon degradation pathways in aquatic sediments. Environ Microbiol.

[bib22] Finke N, Jørgensen BB (2008). Response of fermentation and sulfate reduction to experimental temperature changes in temperate and Arctic marine sediments. ISME J.

[bib23] Finke N, Vandieken V, Jørgensen BB (2007). Acetate, lactate, propionate, and isobutyrate as electron donors for iron and sulfate reduction in Arctic marine sediments, Svalbard. FEMS Microbiol Ecol.

[bib24] Fry JC, Parkes RJ, Cragg BA (2008). Prokaryotic biodiversity and activity in the deep subseafloor biosphere. FEMS Microbiol Ecol.

[bib25] Hedges JI, Keil RG (1995). Marine Chemistry Discussion Paper. Sedimentary organic matter preservation: an assessment and speculative synthesis. Mar Chem.

[bib26] Ho DP, Jensen PD, Batstone DJ (2013). Methanosarcinaceae and acetate-oxidizing pathways dominate in high-rate thermophilic anaerobic digestion of waste-activated sludge. Appl Environ Microb.

[bib27] Holler T, Widdel F, Knittel K (2011). Thermophilic anaerobic oxidation of methane by marine microbial consortia. ISME J.

[bib28] Horsfield B, Schenk HJ, Zink K (2006). Living microbial ecosystems within the active zone of catagenesis: implications for feeding the deep biosphere. Earth Planet Sci Lett.

[bib29] Hubert C, Arnosti C, Bruchert V (2010). Thermophilic anaerobes in Arctic marine sediments induced to mineralize complex organic matter at high temperature. Environ Microbiol.

[bib30] Hubert C, Loy A, Nickel M (2009). A constant flux of diverse thermophilic Bacteria into the cold Arctic seabed. Science.

[bib31] Isaksen MF, Bak F, Jørgensen BB (1994). Thermophilic sulfate-reducing bacteria in cold marine sediments. FEMS Microbiol Ecol.

[bib32] Jiang L, Long C, Wu X (2014). Optimization of thermophilic fermentative hydrogen production by the newly isolated Caloranaerobacter azorensis H53214 from deep-sea hydrothermal vent environment. Int J Hydrogen Energ.

[bib33] Jørgensen BB (1982). Mineralization of organic-matter in the sea bed—the role of sulfate reduction. Nature.

[bib34] Jørgensen BB, Bolin B, Cook RB (1983). Processes at the sediment-water interface. The Major Biogeochemical Cycles and Their Interactions.

[bib35] Joyce AE (2006). The coastal temperature network and ferry route programme: long-term temperature and salinity observations. CEFAS Sci Ser.

[bib36] Kallmeyer J, Boetius A (2004). Effects of temperature and pressure on sulfate reduction and anaerobic oxidation of methane in hydrothermal sediments of Guaymas Basin. Appl Environ Microbiol.

[bib37] Koga Y (2012). Thermal adaptation of the Archaeal and Bacterial lipid membranes. Archaea.

[bib38] Kotelnikova S, Pedersen K (1997). Evidence for methanogenic Archaea and homoacetogenic Bacteria in deep granitic rock aquifers. FEMS Microbiol Rev.

[bib39] Kryachko Y, Dong XL, Sensen CW (2012). Compositions of microbial communities associated with oil and water in a mesothermic oil field. Anton Leeuw.

[bib40] Lipp JS, Morono Y, Inagaki F (2008). Significant contribution of Archaea to extant biomass in marine subsurface sediments. Nature.

[bib41] Lloyd KG, Schreiber L, Petersen DG (2013). Predominant archaea in marine sediments degrade detrital proteins. Nature.

[bib42] Lovley DR, Chapelle FH (1995). Deep subsurface microbial processes. Rev Geophys.

[bib43] Mayumi D, Dolfing J, Sakata S (2013). Carbon dioxide concentration dictates alternative methanogenic pathways in oil reservoirs. Nat Commun.

[bib44] Mayumi D, Mochimaru H, Yoshioka H (2011). Evidence for syntrophic acetate oxidation coupled to hydrogenotrophic methanogenesis in the high-temperature petroleum reservoir of Yabase oil field Japan. Environ Microbiol.

[bib45] Meng J, Xu J, Qin D (2014). Genetic and functional properties of uncultivated MCG archaea assessed by metagenome and gene expression analyses. ISME J.

[bib46] Middelburg JJ, Klaver G, Nieuwenhuize J (1996). Organic matter mineralization in intertidal sediments along an estuarine gradient. Mar Ecol-Prog Ser.

[bib47] Mikucki JA, Liu YT, Delwiche M (2003). Isolation of a methanogen from deep marine sediments that contain methane hydrates, and description of Methanoculleus submarinus sp. nov. Appl Environ Microb.

[bib48] Moreno T, Oldroyd A, McDonald I (2007). Preferential fractionation of trace metals-metalloids into PM10 resuspended from contaminated gold mine tailings at Rodalquilar, Spain. Water Air Soil Poll.

[bib49] Muller AL, de Rezende JR, Hubert CRJ (2014). Endospores of thermophilic bacteria as tracers of microbial dispersal by ocean currents. ISME J.

[bib50] Mussmann M, Brito I, Pitcher A (2011). Thaumarchaeotes abundant in refinery nitrifying sludges express amoA but are not obligate autotrophic ammonia oxidizers. P Natl Acad Sci USA.

[bib51] Muyzer G, Brinkoff T, Nübel U, Akkermans ADL, Van Elsas JD, De Bruijn FJ (1998). Denaturing gradient gel electrophoresis (DGGE) in microbial ecology. Molecular Microbial Ecology Manual.

[bib52] Muyzer G, Dewaal EC, Uitterlinden AG (1993). Profiling of complex microbial-populations by denaturing gradient gel-electrophoresis analysis of polymerase chain reaction-amplified genes-coding for 16S rRNA. Appl Environ Microbiol.

[bib53] Nozhevnikova AN, Nekrasova V, Ammann A (2007). Influence of temperature and high acetate concentrations on methanogenensis in lake sediment slurries. FEMS Microbiol Ecol.

[bib54] O'Sullivan LA, Roussel EG, Weightman AJ (2015). Survival of Desulfotomaculum spores from estuarine sediments after serial autoclaving and high-temperature exposure. ISME J.

[bib55] O'Sullivan LA, Sass AM, Webster G (2013). Contrasting relationships between biogeochemistry and prokaryotic diversity depth profiles along an estuarine sediment gradient. FEMS Microbiol Ecol.

[bib56] O'Sullivan LA, Webster G, Fry JC (2008). Modified linker-PCR primers facilitate complete sequencing of DGGE DNA fragments. J Microbiol Meth.

[bib57] Oremland RS, Marsh LM, Polcin S (1982). Methane production and simultaneous sulphate reduction in anoxic, salt marsh sediments. Nature.

[bib58] Ovreas L, Forney L, Daae FL (1997). Distribution of bacterioplankton in meromictic Lake Saelenvannet, as determined by denaturing gradient gel electrophoresis of PCR-amplified gene fragments coding for 16S rRNA. Appl Environ Microbiol.

[bib59] Parkes RJ, Brock F, Banning N (2012). Changes in methanogenic substrate utilization and communities with depth in a salt-marsh, creek sediment in southern England. Estuar Coast Shelf S.

[bib60] Parkes RJ, Cragg B, Roussel E (2014). A review of prokaryotic populations and processes in sub-seafloor sediments, including biosphere:geosphere interactions. Mar Geol.

[bib61] Parkes RJ, Cragg BA, Banning N (2007a). Biogeochemistry and biodiversity of methane cycling in subsurface marine sediments Skagerrak, Denmark. Environ Microbiol.

[bib62] Parkes RJ, Gibson GR, Mueller-Harvey I (1989). Determination of the substrates for sulfate-reducing bacteria within marine and estuarine sediments with different rates of sulfate reduction. J Gen Microbiol.

[bib63] Parkes RJ, Wellsbury P, Mather ID (2007b). Temperature activation of organic matter and minerals during burial has the potential to sustain the deep biosphere over geological time scales. Org Geochem.

[bib64] Paul K, Nonoh JO, Mikulski L (2012). ‘Methanoplasmatales,’ Thermoplasmatales-related Archaea in termite guts and other environments, are the seventh order of methanogens. Appl Environ Microbiol.

[bib65] Peters V, Conrad R (1996). Sequential reduction processes and initiation of CH4 poduction upon flooding of oxic upland soils. Soil Biol Biochem.

[bib66] Rinke C, Schwientek P, Sczyrba A (2013). Insights into the phylogeny and coding potential of microbial dark matter. Nature.

[bib67] Robador A, Bruchert V, Jørgensen BB (2009). The impact of temperature change on the activity and community composition of sulfate-reducing bacteria in arctic versus temperate marine sediments. Environ Microbiol.

[bib68] Roh Y, Liu SV, Li GS (2002). Isolation and characterization of metal-reducing Thermoanaerobacter strains from deep subsurface environments of the Piceance Basin, Colorado. Appl Environ Microbiol.

[bib69] Rothfuss F, Conrad R (1993). Thermodynamics of methanogenic intermediary metabolism in littoral sediment of Lake Constance. FEMS Microbiol Ecol.

[bib70] Roussel EG, Cambon-Bonavita MA, Querellou J (2008). Extending the sub-sea-floor biosphere. Science.

[bib71] Rui J, Qiu Q, Lu Y (2011). Syntrophic acetate oxidation under thermophilic methanogenic condition in Chinese paddy field soil. FEMS Microbiol Ecol.

[bib72] Schouten S, Middelburg JJ, Hopmans EC (2010). Fossilization and degradation of intact polar lipids in deep subsurface sediments: a theoretical approach. Geochim Cosmochim Acta.

[bib73] Seewald JS (2003). Organic-inorganic interactions in petroleum-producing sedimentary basins. Nature.

[bib74] Stahl DA, de la Torre JR (2012). Physiology and diversity of ammonia-oxidizing Archaea. Annu Rev Microbiol.

[bib75] Stoddard SF, Smith BJ, Hein R (2015). rrnDB: improved tools for interpreting rRNA gene abundance in bacteria and archaea and a new foundation for future development. Nucleic Acids Res.

[bib76] Takai K, Miyazaki M, Hirayama H (2009). Isolation and physiological characterization of two novel, piezophilic, thermophilic chemolithoautotrophs from a deep-sea hydrothermal vent chimney. Environ Microbiol.

[bib77] Valentine DL (2007). Adaptations to energy stress dictate the ecology and evolution of the Archaea. Nature Rev Microbiol.

[bib78] Webster G, Blazejak A, Cragg BA (2009). Subsurface microbiology and biogeochemistry of a deep, cold-water carbonate mound from the Porcupine Seabight IODP Expedition 307. Environ Microbiol.

[bib79] Webster G, Newberry CJ, Fry JC (2003). Assessment of bacterial community structure in the deep sub- seafloor biosphere by 16S rDNA-based techniques: a cautionary tale. J Microbiol Meth.

[bib80] Webster G, Parkes RJ, Cragg BA (2006). Prokaryotic community composition and biogeochemical processes in deep subseafloor sediments from the Peru Margin. FEMS Microbiol Ecol.

[bib81] Webster G, Rinna J, Roussel EG (2010). Prokaryotic functional diversity in different biogeochemical depth zones in tidal sediments of the Severn Estuary, UK, revealed by stable-isotope probing. FEMS Microbiol Ecol.

[bib82] Webster G, Sullivan LA, Meng Y (2015). Archaeal community diversity and abundance changes along a natural salinity gradient in estuarine sediments. FEMS Microbiol Ecol.

[bib83] Wellsbury P, Herbert RA, Parkes RJ (1994). Bacterial [methyl-H-3]thymidine incorporation in substrate-amended estuarine sediment slurries. FEMS Microbiol Ecol.

[bib84] Wery N, Moricet JM, Cueff V (2001). Caloranaerobacter azorensis gen. nov., sp. nov., an anaerobic thermophilic bacterium isolated from a deep-sea hydrothermal vent. Int J Syst Evol Microbiol.

[bib85] Weston NB, Joye SB (2005). Temperature-driven decoupling of key phases of organic matter degradation in marine sediments. P Natl Acad Sci USA.

[bib86] Whiticar MJ (1999). Carbon and hydrogen isotope systematics of bacterial formation and oxidation of methane. Chem Geol.

[bib87] Yanagawa K, Morono Y, de Beer D (2013). Metabolically active microbial communities in marine sediment under high-CO2 and low-pH extremes. ISME J.

[bib88] Zeikus JG, Wolfe RS (1972). Methanobacterium thermoautotrophicus sp n, an anaerobic, autotrophic, extreme thermophile. J Bacteriol.

